# Meniscal repair and partial meniscectomy demonstrated similar clinical outcomes with simultaneous combined anterior cruciate ligament and anterolateral structure reconstruction

**DOI:** 10.3389/fmed.2026.1847295

**Published:** 2026-06-11

**Authors:** Guorui Cao, Xiuli Yang, Xiao Wang, Xiaotao Shi, Lanbo Yang, Peizhao Wang, Honglue Tan

**Affiliations:** Department of Knee Surgery, Luoyang Orthopedic Hospital of Henan Province, Orthopedic Hospital of Henan Province, Luoyang, Henan, China

**Keywords:** anterior cruciate ligament, anterolateral structure, clinical outcome, meniscal repair, meniscectomy

## Abstract

**Purpose:**

The clinical outcomes of patients after combined anterior cruciate ligament (ACL) and anterolateral structure (ALS) reconstruction with or without concomitant meniscal treatment were limited. The purpose of this study was to evaluate clinical outcomes and investigate the effect of concomitant treatment of meniscal injury on these outcomes following combined ACL and ALS reconstruction.

**Methods:**

A total of 86 patients with combined ACL and ALS reconstruction were eligible for inclusion, from August 2018 to November 2022, with at least 1-year follow-up. The patients were assigned to three groups based on meniscal status, including the no injury group (n = 26), the partial meniscectomy group (the meniscus was resected partially, n = 24), and the repair group (the meniscus was sutured, n = 36). Outcome measurements consisted of function, stability, and safety evaluation. Functional evaluation included Lysholm score, Tegner score, and International Knee Documentation Committee (IKDC) score.

**Results:**

At the last follow-up, the Lysholm, Tegner, and IKDC scores were significantly improved compared with preoperative status (*p* < 0.05). Functional scores in the no injury group were much higher than those in the partial meniscectomy and repair groups. In addition, the expense was significantly higher in the repair group (43840.9 ± 10804.9) than that in the no injury (37767.7 ± 4537.4, *p* = 0.003) and partial meniscectomy (37738.7 ± 3794.4, *p* = 0.004) groups. The stability and safety indices did not differ significantly among the three groups (*p* > 0.05).

**Conclusion:**

Among patients following simultaneous ACL and ALS reconstruction with concomitant meniscal injury, meniscal repair and partial meniscectomy could demonstrate comparable functional outcomes.

## Introduction

Anterior cruciate ligament (ACL) injury is a common type of sports injury that is often accompanied by meniscal injury (1). The meniscus, which could withstand compressive force, reduces the impact of exercise on the knee, ensures knee stability, and is an important structure located between the tibial plateau and condyles of the femur (2). The meniscus deficiency could increase the risk of osteoarthritis (OA) and knee laxity (3, 4). Moreover, successful meniscus repair could decrease the risk of graft rupture following ACL reconstruction (5). Therefore, timely meniscus treatment is essential.

ACL reconstruction is the main and standard treatment for ACL injury. Treating injured meniscus concurrently during ACL reconstruction has become a consensus (6–8). The treatment options for meniscus injury include repairing the tear or resecting damaged tissue. Numerous studies have provided evidence indicating that meniscal repair could lead to superior outcomes compared to meniscectomy (1, 9–11). However, some studies have found that no meniscus injury, resection, or repair could obtain equivalent results following ACL reconstruction (12, 13). Furthermore, some authors have confirmed that meniscal repair may result in worse subjective knee function with simultaneous ACL reconstruction (14, 15).

Usually, the patients who underwent additional reconstruction of ALS were associated with a high risk of clinical failure in ACL reconstruction, such as high-grade pivot shift, revision, and generalized ligamentous laxity. Therefore, patients who underwent isolated ACL were significantly different from those who underwent combined ACL and ALS reconstruction. An increasing number of researchers have demonstrated that combined ACL and ALS reconstruction could gain favorable clinical and functional outcomes (16–18). In addition, numerous previous studies have investigated how concomitant treatment of meniscal injury could affect clinical outcomes following isolated ACL reconstruction (6, 11, 15, 19, 20), whereas the study for ACL and ALS reconstruction was still absent. Therefore, we conducted this study to report mid-term clinical outcomes after combined ACL and ALS reconstruction with an autograft and evaluate how concomitant meniscal injury, as well as its surgical method (partial meniscectomy or repair), could affect these outcomes.

## Materials and methods

### Study design and patient’s eligibility for inclusion

This was a retrospective cohort study, and ethical approval for the study protocol was obtained. The patients who underwent combined ACL and ALS reconstruction from August 2018 to November 2022 in our institution were eligible for inclusion. Informed consent to participate was obtained from all the participants in the study. This retrospective study was registered and approved by the Institutional Review Board of Our Hospital (2024XJS0005-02). The study was registered in the ISRCTN80922346; https://www.isrctn.com/login, Registration Date 11 May 2024.

The inclusion criteria were as follows: (1) patients with ACL and ALS reconstruction through a single femoral tunnel, (2) those aged < 50 years, and (3) those with no history of previous ipsilateral knee injury and surgery. The exclusion criteria included: (1) multiple ligament injuries, (2) ACL rupture associated with fracture, (3) ACL revision, (4) significant degree of OA or cartilage damage, and (5) skeletally immature or incomplete medical records. Finally, a total of 86 patients were observed. According to intraoperative meniscus status and surgical method, patients were divided into the no injury group (n = 26), the partial meniscectomy group (the meniscus was resected partially, n = 24), and the repair group (the meniscus was sutured, n = 36).

### Surgical technique

Meniscal injuries were detected at the time of surgery and MRI, and the management included the following: (1) no meniscus injury and subsequently no extra treatment, (2) injury requiring partial meniscectomy, and (3) injury requiring repair. The decision to partially repair or resect the meniscus was made based on the location and type of the injury, the repairability of the tissue, and preoperative discussion with the patient, referring to previous researches (1, 15). Meniscectomy was performed through appropriate arthroscopic scissors and a shaver. Two main suture methods were applied according to the position of the meniscus injury. Regarding the injury located in the anterior horn and body of the meniscus, the suture method was an outside-in technique through a trocar. Regarding injuries located in the posterior root and posterior horn, the suture method was an all-inside technique using a Fast-Fix meniscus suture device (medial meniscus) or a suture hook (lateral meniscus). All tears were sutured tightly with intact meniscal tension.

**Figure 1:**
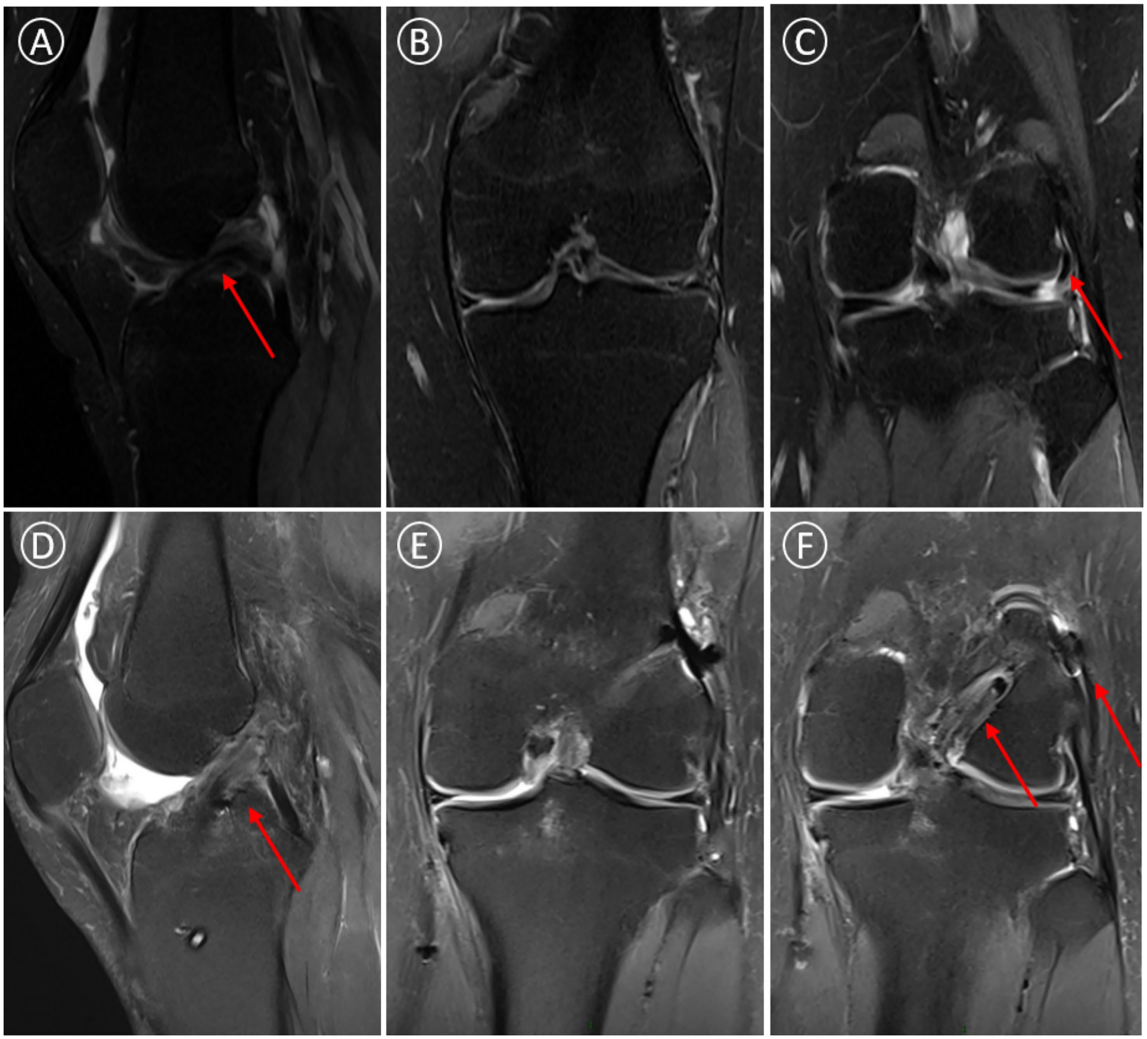
**(A–C)** Images show preoperative magnetic resonance images, **(D–F)** images show postoperative magnetic resonance images of an 18-year-old girl captured at 12 months.

ACL and ALS reconstructions were performed by one experienced surgeon, using autologous gracilis, semitendinosus tendons, and the anterior half of the peroneus longus tendon. Patients underwent additional reconstruction of ALS based on the presence of at least one major criterion (preoperative grade III pivot shift, posterior-inferior tibial slope > 10°, generalized ligamentous laxity or Beighton score ≥4) or at least two secondary standards, including the injury of contralateral ACL, the difference of Lachman test > 7 mm between bilateral knees, irrepairable posterior root injury of the lateral meniscus, combined with Segond fracture, age < 25 years old, and participation in pivoting sports, such as basketball, soccer, and skiing (21, 22). The femoral side was fixed with a hanging titanium plate, and the tibial side was fixed with an absorbable sheathed extrusion screw. ALS and ACL grafts shared the same tunnel on the femoral side. The femoral position (inside out) for ALS was located proximal and posterior to the proximal attachment point of the lateral collateral ligament. The tibial position was the midpoint between the fibular head and Gerdy’s tubercle. Preoperative and postoperative magnetic resonance images are shown in Figure 1.

### Outcome measurements

Outcome measurements included function, stability, and safety evaluation. Functional evaluation included the Lysholm, Tegner, and subjective and objective International Knee Documentation Committee (IKDC) scores. Stability evaluation was performed using the Lachman and Pivot shift test and included potential postoperative complications and clinical failure. The clinical failure of ACL reconstruction was determined by physical examination (Lachman test, anterior drawer test, and pivot shift test) or graft rerupture as detected on imaging or arthroscopic examination according to previous studies (23, 24).

### Statistical analyses

All statistical analyses were performed using SPSS version 25.0 (IBM Corp., Armonk, NY, United States). The continuous variables were compared using the one-way analysis of variance, the Wilcoxon Mann–Whitney U-test, or the independent t-test. The categorical variables were compared using Pearson’s chi-square test or Fisher’s exact test. For the indicators with statistical difference among three groups, a multiple linear regression analysis was performed to establish three regression models. Since the grouping variable consisted of three categories (no meniscus injury, meniscectomy, and repair), dummy variables were generated prior to the regression analysis. No meniscus injury group served as the reference group, and dummy variables were subsequently created for partial meniscectomy group and repair group. Statistical significance was considered at a *p*-value of < 0.05.

## Results

### Patients’ demographics

A total of 103 patients who underwent combined ACL and ALS reconstruction were eligible for inclusion in this study. Subsequently, 17 patients were excluded according to the exclusion criteria, and 86 patients (26 in the no-injury group, 24 in the partial meniscectomy group, and 36 in the repair group) were observed and studied. The body mass index (BMI) was higher in the partial meniscectomy (26.6 ± 4.7) and repair (26.2 ± 3.8) groups than that in the no injury group (23.3 ± 2.2, *p* = 0.042). Motor vehicle accidents were the secondary cause of injury in the repair group, whereas no patient in the other two groups had experienced motor vehicle accidents (*p* = 0.018). In addition, the operating time in the repair group was the longest among the three groups (*p* < 0.001). The descriptive characteristics are summarized in Table 1.

**Table 1:** Baseline characteristics.

Baseline characteristic	No meniscus injury group (*n* = 26)	Partial meniscectomy group (*n* = 24) (*n* = 50)	Repair group (*n* = 36)	P
Age (y)	27.0 ± 6.5	30.6 ± 8.0	28.8 ± 9.4	0.610
Sex (male/female)	20/6	20/4	26/10	0.608
BMI(kg/ $m^{2}$ )	23.3 ± 2.2	26.6 ± 4.7	26.2 ± 3.8	0.042*
Side, left: right	10/16	8/8	18/18	0.135
Generalized ligamentous laxity (%)	8 (30.7%)	6 (25.0%)	16 (44.4%)	0.263
Reason of injury (%)				0.018*
Sports injury	22	16	25	
Auto accident	0	0	7	
Fall injury	2	6	3	
Other	2	2	1	
Anesthesia method				0.311
General	6	8	15	
Regional	20	16	21	
ASA class				0.513
1–2	25	24	34	
≥3	1	0	2	
Follow-up (month)	29.7 ± 9.5	29.6 ± 10.3	26.1 ± 10.7	0.515
Operating time (min)	95.4 ± 23.0	97.5 ± 19.4	124.2 ± 30.2	<0.001*

BMI, Body mass index = weight/height^2^; ASA, American Society of Anesthesiologists. *Significant difference.

### The comparison of preoperative and postoperative outcomes at the latest follow-up

**Figure 2:**
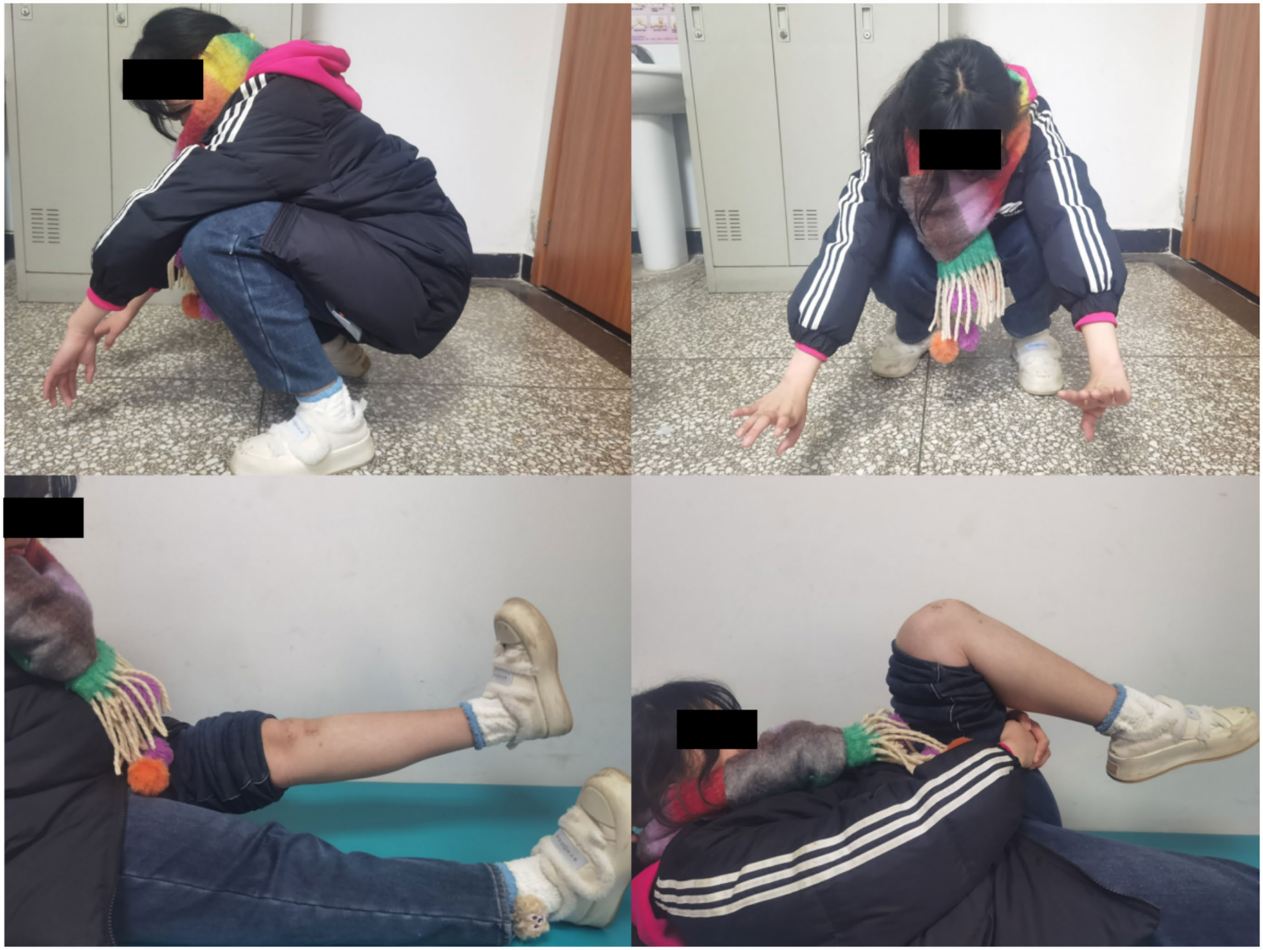
Postoperative functional recovery image of an 18-year-old girl at 12 months after combined ACL and ALS reconstruction.

At the latest follow-up, the Lysholm, subjective IKDC, and Tegner scores were significantly improved compared to their preoperative status (*p*<0.05). Besides, the index of objective IKDC score was markedly ameliorated (*p*<0.05). The results are shown in Table 2 and Figure 2.

**Table 2:** Comparison of clinical outcomes pre- and post-operatively at latest follow-up.

Variable	Preoperative (*n* = 86)	Postoperative (*n* = 86)	P
Lysholm score	64.8 ± 20.9	94.0 ± 6.6	<0.001*
Tegner score	2.2 ± 1.3	5.9 ± 1.4	<0.001*
Subjective IKDC score	51.0 ± 21.2	89.5 ± 10.0	<0.001*
Objective IKDC score (A/B/C/D)	0/21/46/19	80/5/1/0	<0.001*

IKDC, International Knee Documentation Committee Subjective Knee Form. *Significant difference.

### Comparison of clinical outcomes

The subjective IKDC score was higher in the no injury group (94.0 ± 6.1) than that in the partial meniscectomy (87.7 ± 9.7, *p* = 0.037) and the repair (87.4 ± 11.5, *p* = 0.017) groups. Similarly, the Tegner score was higher in the no injury group (6.6 ± 1.4) than that in the partial meniscectomy (5.5 ± 1.2, *p* = 0.008) and the repair (5.6 ± 1.4, *p* = 0.006) groups. The difference between the partial meniscectomy group and the repair group was not significant. In addition, the expense was significantly higher in the repair group (43,840.9 ± 10,804.9) than that in the no injury (37,767.7 ± 4537.4, *p* = 0.003) and the partial meniscectomy (37,738.7 ± 3,794.4, *p* = 0.004) groups. However, the differences in objective IKDC score, pivot shift test, and Lachman test among the three groups did not reach statistical significance (*p*>0.05). Related results are summarized in Table 3.

**Table 3:** Comparison of patient-reported related outcomes.

Variable	No injury group (*n* = 26)	Partial meniscectomy group (*n* = 24)	Repair group (*n* = 36)	P	P1	P2	P3
Lysholm score	96.0 ± 3.7	93.2 ± 6.7	93.1 ± 7.8	0.228	–	–	–
Subjective IKDC score	94.0 ± 6.1	87.7 ± 9.7	87.4 ± 11.5	0.036*	0.037*	0.017*	0.911
Tegner score	6.6 ± 1.4	5.5 ± 1.2	5.6 ± 1.4	0.009*	0.008*	0.006*	0.834
Objective IKDC score (A/B/C/D)	26/0/0/0	22/1/1/0	32/4/0/0	0.188	–	–	–
Pivot shift test (0/1/2/3)	26/0/0/0	23/0/1/0	35/1/0/0	0.407	–	–	–
Lachman test (0/1/2/3)	25/1/0/0	22/1/1/0	34/2/0/0	0.605	–	–	–
Expenses ∆	37767.7 ± 4537.4	37738.7 ± 3794.4	43840.9 ± 10804.9	0.002*	0.989	0.003*	0.004*

IKDC, International Knee Documentation Committee. P represents the P-value of no injury vs. meniscectomy vs. repair group, P1 represents the p-value of no injury vs. the meniscectomy group, P2 represents the p-value of no injury vs. the repair group, and P3 represents the p-value of meniscectomy vs. the repair group. ∆Results were presented as Chinese yuan. *Significant difference.

### Results of the multivariate regression analysis

For the subjective IKDC and Tegner scores, a regression analysis revealed that the direction of the beta coefficient was consistent and stable across the three models. For the expenses, the *p*-value for the dummy variable of group 2 was not statistically significant in any of the three models (*p* > 0.05). For the group 3 dummy variable, the direction of the beta coefficients was consistent and stable across the three models. Related results are summarized in Table 4.

**Table 4:** Results of the multivariate regression analysis.

Item	Model 1		Model 2		Model 3	
	Beta (95% CI)	*p* value	Beta (95% CI)	*p* value	Beta (95% CI)	*p* value
Subjective IKDC score group 2	−6.33 (−12.27, −0.38)	0.037*	−6.12 (−12.29, 0.05)	0.042*	−7.49 (−13.40, −1.59)	0.014*
Subjective IKDC score group 3	−6.64 (−12.04, −1.24)	0.017*	−6.42 (−12.09, −0.75)	0.027*	−5.07 (−10.51, 0.37)	0.067
Tegner score group 2	−1.14 (−1.97, −0.31)	0.008*	−0.97 (−1.81, −0.13)	0.024*	−1.28 (−2.12, −0.45)	0.003*
Tegner score group 3	−1.06 (−1.80, −0.31)	0.006*	−0.90 (−1.66, −0.13)	0.022*	−0.97 (−1.64, −0.10)	0.028*
Expenses group 2	−29.01 (−4369.01, 4310.99)	0.989	−101.61 (−4725.05, 4521.83)	0.965	645.36 (−3802.55, 5093.27)	0.774
Expenses group 3	6073.20 (2127.22, 10019.18)	0.003*	6008.88 (1816.67, 10201.08)	0.006*	5863.60 (1695.03, 10032.15)	0.006*

Group 1: no meniscus injury group; group 2: partial meniscectomy group; group 3: repair group. Model 1 is an unadjusted model that includes only the grouping variable. Model 2 includes BMI as a covariate based on model 1. Model 3 included the mechanism of injury as a covariate based on model 1.

### Complications

There was one patient in the partial meniscectomy group and one patient in the repair group who developed deep vein thrombosis (*p* = 0.604). One patient in the no injury group and one patient in the repair group were diagnosed with knee joint stiffness and subsequently underwent a release operation (*p* = 0.648). Two patients suffered from wound complications. Moreover, two patients (one patient in the partial meniscectomy group and another in the repair group) still had grade II pivot shift at the latest follow-up, regarded as clinical failure. The incidence of complications showed no statistical difference among the three groups (*p*>0.05; Table 5). All patients were discharged uneventfully after symptomatic treatment.

**Table 5:** Complications.

Complications	No meniscus injury group (*n* = 26)	Partial meniscectomy group (*n* = 24)	Repair group (*n* = 36)	P
PE	0	0	0	–
DVT	0	1	1	0.604
Knee joint stiffness	1	0	1	0.648
Wound complication	0	1	1	0.604
Clinical failure	0	1	1	0.604

PE, Pulmonary embolism; DVT: deep venous thrombosis.

## Discussion

Sporting activity and related injuries have been increasing year by year. ACL injury is one of the most common knee sports injuries (25). Arthroscopic ACL reconstruction with autologous tendon is a mainstream therapeutic method (26). However, isolated ACL reconstruction is not enough to restore rotational stability of the knee, particularly for patients with a high risk of failure in ACL reconstruction, such as high-grade pivot and generalized ligamentous laxity. Combined ACL and ALS reconstructions could significantly improve knee function and stability without increasing the related risk in patients with a high risk of failure in ACL reconstruction (16–18). In addition, it has been reported that ALS appeared to confer a protective effect on the meniscus repair performed at the time of ACL reconstruction. Combined ACL and ALS reconstruction could reduce the risk of reoperation for failure (27). Simultaneous treatment of the meniscus is a vital procedure of ACL reconstruction. Abundant previous literature focuses on the meniscus management following primary ACL reconstruction (4, 6, 8, 13, 19, 28), while the research on patients with combined ACL and ALS reconstruction is still lacking. To the best of our knowledge, this is the first study to compare the influence of different meniscus management approaches (no meniscus operation, partial meniscectomy, and repair) on clinical outcomes following ACL and ALS reconstruction. The aim of this study is to report clinical outcomes following combined ACL and ALS reconstruction and to evaluate how concomitant meniscal injury, as well as its surgical method (partial meniscectomy or repair), could affect related clinical outcomes. We found that patients without meniscus injury had superior clinical outcomes. Meniscus repair and partial meniscectomy with simultaneous ACL and ALS reconstruction could result in equivalent clinical outcomes.

The risk of concomitant meniscal tear in the occurrence of ACL injury is substantial. Several studies have investigated the effect of clinical outcomes with or without incidental meniscus injury following primary ACL reconstruction (13, 15, 20, 28). A large sample study enrolled 6,398 patients with primary ACL reconstruction, demonstrating that patients with concomitant meniscal resection were able to reach the same subjective knee function as those with no meniscus injury; however, patients with meniscal repair had slightly worse results (15). Singh et al. drew a similar conclusion, revealing that there was no significant difference in postoperative outcome scores between patients with or without meniscal injury at a mean follow-up of 3.5 years (20). Another large sample study, including 5,378 patients with primary ACL reconstruction, found that patients without meniscal injury and those with meniscal resection or repair could lead to equivalent clinical results (13). Phillips et al. held a contradictory view. Their study indicated that meniscus meniscectomy resulted in worse clinical outcomes, as patients without concomitant meniscus injury in addition to ACL reconstruction, at 2-year follow-up (28). Currently, information on the literature is insufficient to evaluate how incidental meniscus injury affects knee function after combined ACL and ALS reconstruction. In our study, we found that compared with patients with meniscus meniscectomy or repair, patients without meniscus injury have superior clinical outcomes following combined ACL and ALS reconstruction.

The selection between meniscectomy and meniscus repair during ACL reconstruction depends on the condition of meniscal tears and their potential for successful repair. Numerous studies have provided evidence indicating superior outcomes associated with meniscus repair compared to partial meniscectomy (1, 6, 9–12). However, some authors held different views. Casp et al. found that persistent weakness, asymmetry, and reduced subjective outcome scores were not influenced by meniscal treatment after ACL reconstruction. Partial meniscectomy and meniscus repair resulted in comparable patient-reported outcomes (12). In addition, Svantesson et al. have demonstrated that patients with meniscal repair may have slightly worse subjective knee function at both 6- and 12-month follow-up (15). However, the follow-up time of the above studies was relatively short, not exceeding 1 year. Moretti et al. demonstrated that biomechanical performance measures and fear of reinjury were significantly worse with associated meniscal repair, especially in those with a lateral meniscal tear. Their research object was soccer players, not suitable for everyone (14). The abovementioned studies concentrated on the relationship between simultaneous meniscus injury and primary ACL reconstruction. Our study indicates that patients with meniscus repair and partial meniscectomy have equivalent functional outcomes following combined ACL and ALS reconstruction.

Previous studies have indicated that higher BMI may be associated with an increased prevalence of concomitant meniscal tears in ACL injury (29–31). Perkins et al. have shown that pediatric patients undergoing ACL reconstruction had a 58% incidence of concomitant meniscal injury, and increasing BMI was an independent risk factor for these injuries (30). Similarly, two additional studies demonstrated that higher BMIs were found to have an increased risk of meniscus injury among patients undergoing ACL reconstruction (29, 31). In this study, we found that the BMI in the partial meniscectomy and repair groups was much higher than that in the no meniscus group following ACL and ALS reconstruction. Therefore, when reconstructing the ACL for patients with high BMI (>26 kg/ $m^{2}$ ), carefully examining to detect possible meniscus injury is very important.

Our study showed that the hospitalization expense of meniscus repair is much greater than that of partial meniscectomy in patients who underwent ACL and ALS reconstruction (43840.9 ± 10804.9 vs. 37738.7 ± 3794.4, *p* = 0.004). Similarly, previous studies have found that meniscus repair has a higher initial expense and failure rate than partial meniscectomy. However, for long-term costs and effects, meniscus meniscectomy could increase the risk of loss of meniscal function, accelerate articular cartilage deterioration, and even OA (32, 33). Moreover, some authors have demonstrated that meniscus repair, compared with meniscectomy and non-surgical treatment, could reduce OA and total knee arthroplasty incidence, as well as save substantial costs. Therefore, meniscal repair at the time of ACL reconstruction was more cost-effective than partial meniscectomy (34–36).

This study has several limitations. First, the specific location and types of meniscus injury, such as medial or lateral, anterior or posterior horn, and horizontal, vertical, or longitudinal tear, were not clearly classified or analyzed. In addition, we did not compare the discrepancy of different meniscus suture methods. Second, the data of the second arthroscopy were absent; therefore, the actual meniscus healing after meniscal repair lacked an intuitive basis. Third, the duration of follow-up was not long enough to detect potential degenerative changes, such as knee OA and knee laxity. It was reported that partial meniscectomy was the main risk factor for long-term OA development (3). Fourth, the baseline characteristics were not completely consistent. BMI is significantly higher in the partial meniscectomy and repair groups. The reason for the injury is also different. The otherness of BMI and the mechanism of injury among the three groups may affect clinical outcomes. The adjustment for confounding variables was absent. Fifth, the uniform standard for patients who underwent combined ACL and ALS reconstruction across all patients was not clearly defined, which raised concerns about the cohort consistency. In addition, all surgeries were performed by a single experienced surgeon, which may limit the generalizability of the findings. Last but not the least, this study was a retrospective study with a small sample size. A retrospective study used the existing historical data. The data might be missing, inaccurate, or inconsistent, which increases the risk of bias. Future study with high-quality evidence was necessary.

## Conclusion

Among patients following simultaneous ACL and ALS reconstruction with concomitant meniscal injury, meniscal repair and partial meniscectomy could demonstrate comparable functional outcomes.

## Data Availability

The original contributions presented in the study are included in the article/supplementary material; further inquiries can be directed to the corresponding author/s.
